# Suppressive and additive effects in protection mediated by combinations of monoclonal antibodies specific for merozoite surface protein 1 of *Plasmodium yoelii*

**DOI:** 10.1186/1475-2875-9-46

**Published:** 2010-02-10

**Authors:** Irosoki Eslava, Gilberto Payares, Beatriz M Pernia, Anthony A Holder, Lilian M Spencer

**Affiliations:** 1 School of Bioanalysis, Central University of Venezuela, Caracas, Venezuela; 2 Institute of Experimental Biology, Central University of Venezuela, Caracas, Venezuela; 3 Department of Cell Biology, Simón Bolívar University, AP 89000, Caracas, Venezuela; 4 Division of Parasitology, MRC National Institute for Medical Research, Mill Hill, London, UK

## Abstract

**Background:**

The merozoite surface protein (MSP)-1 is a target antigen of protective immunity and a malaria vaccine candidate. The nature of this protective immune response warrants further investigation: although specific antibody is thought to play a major role, the mechanisms of protection are still unclear. Monoclonal antibodies (mAbs) specific for the C-terminus of MSP-1 from *Plasmodium yoelii *have been shown previously to provide protection against challenge infection when administered by passive immunization to mice. Three protective mAbs were re-examined and, in particular, the effect of combinations of antibodies on the protection provided was studied. It was found that a combination of two antibodies can either provide additive protective effects or result in a suppression of protection. In this report the importance of antibody subclass and epitope specificity in the outcome of these passive immunization experiments are discussed.

**Methods:**

The minimum protective dose (MPD) for each mAb was determined, and then combinations of antibody at their MPD were investigated for their ability to control parasitaemia and promote survival in groups of mice. Mice were inoculated over three days with the MPD and challenged with a blood stage infection of the virulent *P. yoelii *17 XL. The resultant parasitaemia was assessed daily on Giemsa-stained blood films. Following the infection the presence of MSP-1 specific antibodies in the sera was monitored, and the proliferative responses of cells in the spleen of protected mice were measured.

**Results:**

Combining antibodies resulted in either an additive effect on protection, with reduced peak parasitaemia and better survival, or resulted in a suppression of protection over that achieved by a single antibody alone. An additive effect was observed when B6 and F5 that have the same isotype and similar fine specificity, were combined. However, a combination of mAb D3, an IgG2a, with either B6 or F5 (both IgG3) suppressed protection, an effect that may have been due to the combination of different isotypes or to the different fine specificity of the antibodies.

**Conclusions:**

These results suggest that a combination of protective antibodies with either the same or different isotypes can produce either an additive or a suppressive effect in passive immunization. This phenomenon may be important in better understanding immunity in this experimental mouse model of malaria.

## Background

Malaria control remains one of the most important priorities for improving public health in tropical and subtropical areas of the world. The World Health Organization estimates that half the world's population is at risk and there are about 250 million clinical cases in Africa, Asia and South-America, with up to a million deaths a year due to malaria [1]. The malaria parasite has evolved complex strategies to adapt to its host and evade the immune system.

Recent studies on immunity to malaria have been largely carried in the field using human material or in the laboratory using appropriate murine models. A large number of parasite proteins have been studied as antigens, including the merozoite surface protein (MSP)-1 [2]. The merozoite is a specialized cell that invades red blood cells, representing an essential extracellular stage of the asexual blood cycle. Proteins on the merozoite surface are, therefore, accessible to humoral immunity and there is considerable interest in understanding how antibodies binding to these proteins can either prevent erythrocyte invasion or target merozoites for phagocytosis and clearance. MSP-1 is a high-molecular-weight protein synthesized as a precursor during schizogony, which is found on the surface of the merozoite as a complex of fragments derived by proteolytic processing of the precursor [2,3]. There is abundant evidence to suggest that the C-terminal region of this molecule, represented by a 42 kDa fragment on the merozoite surface (MSP1_42_), is the target of antibodies that are important in protective immunity [4]. At invasion this fragment is further cleaved into two further fragments, one of which is the C-terminal 19 kDa fragment (MSP-1_19_) that is comprised of two epidermal growth factor-like domains and remains on the surface of the parasite through erythrocyte invasion.

In the present work, three monoclonal antibodies (mAbs) that bind to the C-terminus of *Plasmodium yoelii *MSP-1 were used, which all individually provide protection against a challenge infection following passive immunization of mice [5]. The three antibodies that have both different and similar fine specificities and isotypes, are D3 (IgG2a), F5 (IgG3) and B6 (IgG3). B6 and F5 both bind to the first epidermal growth factor (EGF) domain in MSP-1_19 _with a similar fine specificity, whilst D3 binds to an epitope that is only found on the intact MSP-1_42_. The aim of this study was to investigate the possible suppressive, synergistic or additive effects of combining these mAbs in passive immunization experiments using groups of BALB/c mice.

## Methods

### Monoclonal antibodies (mAbs)

Hybridomas expressing the mAbs B6 (IgG3), D3 (IgG2a) and F5 (IgG3) were maintained and cultured as described previously [5]. Immunoglobulins were purified from hybridoma culture supernatants using protein G-Sepharose according to the manufacturer's recommendations.

### Passive immunization and parasite challenge

Eight week-old female BALB/c mice bred under specific-pathogen-free conditions were used in groups of six. The purified mAbs dissolved in phosphate buffered saline (PBS) were administered by intraperitoneal injection on three occasions, i.e. one day before, one day after and on the day of challenge infection. The parasite challenge was administered by intravenous injection of five thousand parasitized erythrocytes, at least one hour after administration of the mAb. The parasite used for the challenge was the lethal 17XL strain of *P. yoelii*; the parasite was stored at - 80°C and passaged once in a mouse before use in these experiments. Blood stage parasitaemia was assessed daily on smears made from tail blood and stained with Giemsa's reagent.

The amount of antibody to be used in the passive immunization experiments was determined in preliminary experiments carried out to establish the minimum protective dose (MPD), using a range down to one fifth of the 1.5 mg amount used previously [5]. All animal experimentation was approved by the Ethical Review board of USB.

### Western blot assay

To detect antibodies to MSP-1 in the sera from the immunized mice, a western blot assay was used. A recombinant protein comprised of glutathione S-transferase fused to the two C-terminal MSP-1 epidermal growth factor-like domains (GST-MSP-1_19_) was expressed in *Escherichia coli *and purified from the bacterial lysates as described previously [6]. The purified protein was subjected to SDS-PAGE and then electrophoretically transferred to nitrocellulose paper (NCP, 0.2 μm pore size; Schleicher & Schuell), at 120 mA in a transblotting chamber (Bio-Rad, Instruments), for one hour at 4°C, using 25 mM Tris-HCl, 150 mM glycine, 20% v/v methanol, by the method of Towbin and co-workers [7]. After transfer, the blots were blocked by incubation with a solution of 3% w/v non-fat milk in PBS for 30 min at room temperature, and washed three times (3×) in PBS, containing 0.05% v/v Tween-20. Blots were then incubated for one hour at room temperature with a solution of primary antibody diluted 1:200 in PBS, washed 3× and incubated in a solution of affinity purified goat anti-mouse immunoglobulin conjugated to horseradish peroxidase, at a 1:1,000 dilution (Sigma), for a further one hour. The blots were washed again 3 times and antibody binding was detected by incubation in a solution of 3 mg ml^-1 ^4-chloro-1-napthol in methanol, mixed with 50 ml of 50 mM Tris-HCl pH 7.5, and 30 μL of 30% H_2_O_2_. The colour reaction was stopped by washing with H_2_O.

### Cell proliferation assay

Cells were harvested from the spleens of naïve mice or surviving mice in the group that had been passively immunized with the combination of B6 and F5 mAbs, and placed into culture in sterile 96-well tissue culture plates with minimum essential medium (MEM) supplemented with 20 mM L-glutamine, 16.5 mM NaHCO3, 10% v/v foetal calf serum and 2% antibiotic (penicillin/streptomycin) solution. A suspension of 2 × 10^5 ^cells was placed in each well with 10 or 20 μg of either recombinant MSP-1_19 _protein or concanavalin A (ConA) as a lymphocyte mitogen control, or buffer alone as a negative control. After 48 hours of incubation at 37°C and in an atmosphere of 5% v/v CO_2 _in air, cell viability and proliferation was assessed spectrophotometrically following the addition of 20 μl of MTS reagent ([3-(4,5-dimethylthiazol-2-yl)-5-(3-carboxymethoxyphenyl)-2-(4-sulfophenyl)-2H-tetrazolium, inner salt; MTS^(a)^], CellTiter 96^® ^AQueous, Promega). The reduction in absorbance at 490 nm for the treated and untreated control cultures was measured and compared.

### Statistical analysis

Each experimental value is presented as the mean of six replicates ± standard deviation. Once normality and homogeneity criteria were satisfied, statistical analyses were carried out by one-way ANOVA, taking α = 5% (p < 0.05) as significant.

## Results

**Figure 1:**
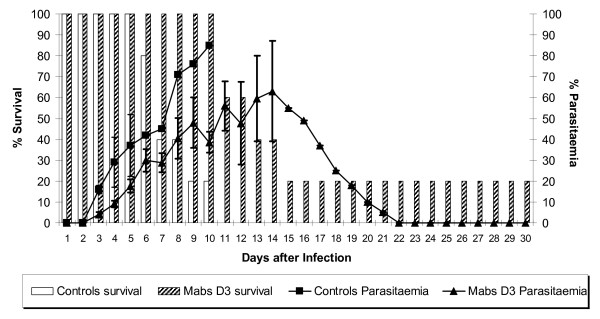
**Passive immunization with the minimal protective dose (MPD) of mAb D3**. The bars represent the percentage survival and the lines the percentage parasitaemia for the control group of animals and the group receiving mAb D3.

In the first series of studies, the MPD was established for each mAb. For each of the three mAbs a total dose of 0.3 mg (i.e. 0.1 mg delivered on three occasions) modulated the course of infection but was not protective. At a higher dose (0.6 mg of mAb D3; 0.9 mg of B6 and 1.2 mg of F5) a clear protective effect was seen for each of the antibodies (data not shown). In the control group inoculated with PBS alone a fulminating infection resulted in high parasitaemia with no survival past day 10. In contrast passive immunization with 0.6 mg of mAb D3 delayed the course and reduced the peak of parasitaemia as well as improving survival (Figure 1). All animals were alive on day 10 and 20% survived to the end of the experiment on day 30. There was a 2-day delay in patent parasitaemia, which then increased to 63% only by day 14 followed by clearance of parasites by day 22. The difference in parasitaemia between experimental and control group, for example at day 9, was statistically significant.

**Figure 2:**
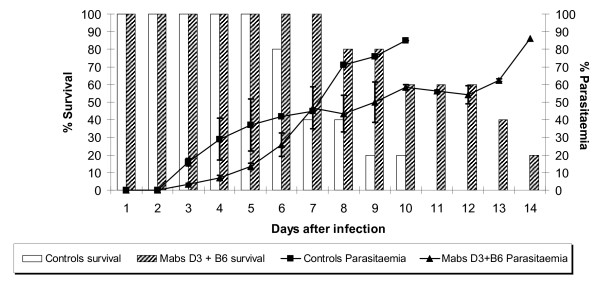
**Passive transfer experiment with the mAbs D3 and B6 in combination**. The bars represent the percentage of survival and the lines the percentage of parasitaemia.

**Figure 3:**
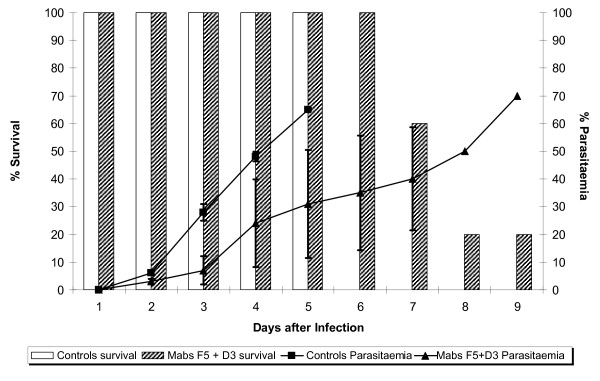
**Passive transfer experiment with the mAbs D3 and F5 in combination**. The bars represent the percentage of survival and the lines the percentage of parasitaemia.

Then, the consequence of passive immunization with mixtures of the mAbs at their MPD was examined, expecting an additive effect. The effect of immunization with a combination of D3 and B6 mAbs is shown in Figure 2. As can be seen from this graph, passive immunization with a mixture of these antibodies produced a lower level of protection than that achieved with individual mAbs alone. There was a protective effect when the survival rate and parasitaemia were compared to that in the control group injected with PBS alone in which group there was nearly 90% parasitaemia and no survival beyond day 10. However, parasite development in the presence of this combination resulted in both higher mortality (20% survival by day 14) and parasitaemia (85% on day 14) when compared with the protection given by B6 (57% parasitaemia) or D3 (63% parasitaemia) alone. Thus the suppressive effect on parasitaemia of the combination was less than that achieved by the individual antibodies given separately. A similar suppressive effect was observed in a passive immunization experiment using a combination of the D3 and F5 mAbs. As shown in Figure 3, the combination of D3 and F5 resulted in 70% parasitaemia at day 9 compared to 63% and 66% for D3 and F5 alone, respectively. On this occasion, none of the control mice survived beyond day 5 with a 70% parasitaemia, consistent with a higher parasite challenge inoculum.

**Figure 4:**
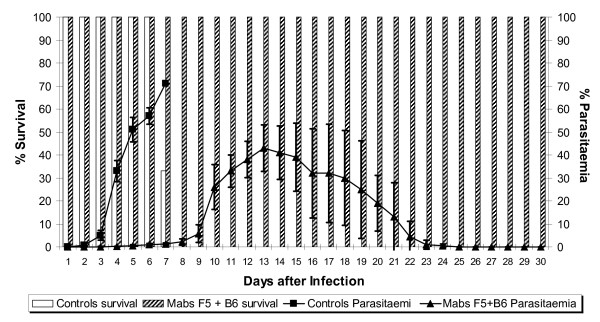
**Passive transfer experiment with the mAbs B6 and F5 together in combination**. The bars represent the percentage of survival and the lines the percentage of parasitaemia.

The third possible combination of antibodies was mAbs B6 with F5, both of which are of the IgG3 isotype. In this case the outcome of the passive immunization with the combination was a clear additive effect, as shown in Figure 4. In this experiment none of the control group survived beyond day 7 and with a parasitaemia greater than 70%. In contrast all the mice that received this combination of antibodies survived to the end of the experiment at day 30 with no detectable parasites after day 23. Furthermore the maximum parasitaemia was 43%, considerably lower than that in the groups given mAbs B6 and F5 separately. In these passive immunization experiments, from day 2 there was a significantly lower parasitaemia (p ≤ 0.05) than in the control group. In order to confirm that the mice from the group protected by passive immunization with B6 and F5 did not have subpatent parasites after clearance of parasitaemia, a 200 μl blood sample was taken and inoculated intravenously into a naive mouse; no parasites were detected in these animals during the 30-day monitoring period.

**Figure 5:**
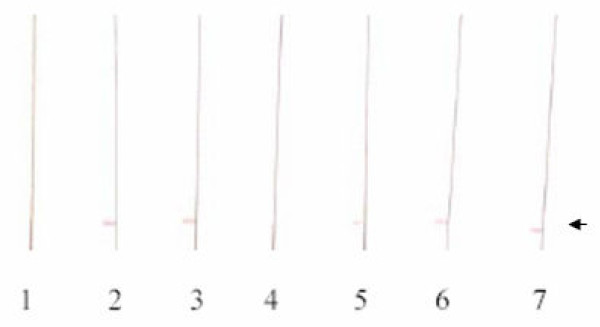
**Western blot analysis of the sera from the surviving animals from the passive transfer experiments (PTE)**. (1). Normal mouse serum, (2). Serum from PTE of D3+B6, (3). Serum from PTE of D3+F5, (4). Serum from PTE of B6+F5, (5). Serum from PTE of D3, (6). Serum from PTE of B6, (7). Serum from PTE of F5. The arrow indicates the location of the recombinant 46 kDa GST-MSP-1_19 _protein.

To confirm the presence of antibodies to MSP-1 in the surviving animals from the different groups, serum samples were examined by western blotting against a GST-MSP1_19 _fusion protein. Antibodies were detected in all of the serum samples from mice that had been administered the mAbs either alone or in combination except for the group that received a mixture of B6 and F5 mABs (Figure 5).

To examine whether or not passive immunization and challenge infection induced an immune response, splenic lymphocytes from the group immunized with the combination of B6 and F5 antibodies were examined for their ability to proliferate in the presence of recombinant MSP-1, as shown in Table 1. Lymphocyte proliferation in the presence of recombinant MSP-1 protein was similar to that in the presence of the mitogen Con A, and much higher than the proliferative response to MSP-1_19 _of lymphocytes from naïve mice, indicating that an immune response had been induced in these protected animals.

**Table 1:** Cell proliferation assay following recovery from infection.

Spleen cell source	Stimulant	Amount (μg)	Absorbance
**Normal**	MSP-1_19_	10	0.090
**Normal**	MSP-1_19_	20	0.072
**F5+B6 recovered**	Con A	10	0.302
**F5+B6 recovered**	Con A	20	0.329
**F5+B6 recovered**	MSP-1_19_	10	0.338
**F5+B6 recovered**	MSP-1_19_	20	0.445

Normal spleen corresponds to cells from naïve mice as negative control and cells stimulated with Con A are a positive control of proliferation/viability, detected in the MTS assay. A blank value of 0.147, obtained with normal spleen cells without stimulation with MSP-1_19 _or ConA, was subtracted from each absorbance value. Each absorbance value represents the mean from three independent experiments.

## Discussion

It is well established that immunization of mice with the C-terminal region of MSP-1 is able to induce protective immunity [6,8,9]. What is less clear are the protective mechanisms responsible. There is good evidence that antibody plays an important role in the protection induced [5,6,10], but the whether the antibody works by directly neutralizing merozoites and blocking erythrocyte invasion or primarily through Fc-mediated actions needs further investigation.

The ability of three monoclonal antibodies specific for the merozoite surface protein MSP-1 to provide protection against infection with the rodent malaria parasite *P. yoelii *by passive immunization was investigated. In particular we have examined the effect of combining pairs of antibodies on their protective capacity. These antibodies are of the G3 (B6 and F5) and G2a (D3) isotypes. Both B6 and F5 bind to an epitope within the first EGF-like domain of *P. yoelii *MSP-1_19_, whereas D3 binds to an epitope in the longer MSP1_42 _fragment, which is formed from the two subdomains of MSP1_33 _and MSP1_19 _[5]. The epitopes binding B6 and F5 overlap but are not identical [5,11], but their proximity to the epitope of D3 is unclear since the epitope for this antibody appears to be a conformational epitope that is formed only in the intact MSP-1_42_. In the first step of this study, the minimum protective dose (MPD) was determined for each antibody as 0.6, 0.9 and 1.2 mg for mAbs D3, B6 and F5, respectively. In the second step of the study, the ability of combinations of two antibodies at their MPD to provide protection by passive immunization was measured. Mixtures of D3 and B6 or D3 and F5 antibodies were less effective than the single antibodies alone, whereas a mixture of F5 and B6 was more effective than either of these two antibodies alone.

The mechanism(s) by which these three antibodies provide protection following passive immunization is unknown. There are several mechanisms that have been suggested to explain the protective effect of antibodies to merozoite surface proteins [12], and a number of possible mechanisms have been identified for antibodies directed against the C-terminal region of *Plasmodium falciparum *MSP-1 [4]. These mechanisms may depend on the antibody alone, such as inhibition of proteolytic processing of MSP-1 or merozoite agglutination, or depend on an Fc-mediated component such as opsonisation of merozoites and phagocytosis. In both types of mechanism it is likely that the antibody avidity and concentration are important. In the case of antibody mediated inhibition of invasion through inhibition of MSP-1 processing the fine specificity of antibody binding is crucial. Antibodies that bind to the first EGF-like domain, such as B6 and F5 can inhibit processing but the importance of this mechanism in protection against *P. yoelii *has not been established. If both B6 and F5 mAbs are providing protection through this mechanism, it is not surprising that the protection provided by passive immunization with a mixture of the two is additive since this is equivalent to increasing the concentration of the protective antibody specificity. In fact, the mice in this group fully resolved their parasitaemia since a blood sample that was transferred from each mouse into naïve recipient mice failed to cause infection. However, it is also possible that an acquired immune response as indicated by the lymphocyte proliferation (Table 1) and resulting from the parasite infection may have also contributed to this clearance.

The failure of mixtures of B6 with D3 or F5 with D3 to provide additive protection is more difficult to explain. mAb D3 does not bind to MSP1_19 _alone and yet is very effective at providing protection on passive immunization; a corresponding antibody specific for *P. falciparum *MSP-1 has not been described or a mechanism evaluated. The suppressive effect of the mixture on the control of parasitaemia highlights the potential complexity of antibody interactions within a polyclonal response. It has already been established that some antibodies, which do not inhibit MSP-1 processing, can be defined as blocking antibodies in this mechanism because they block the binding of the inhibitory antibody [4,13,14]. It is not known whether or not the binding of D3 to MSP-1 competes with and blocks the binding of B6 or F5 mAbs, or *vice versa*. In either case the consequence may be a reduction in the protective capacity of the mixture of antibodies, as seen in the results presented here.

An alternative explanation may reflect the importance of Fc-mediated mechanisms in the protection mediated by passive immunization with antibodies to MSP-1, which may depend on the isotype of the mAb. It has already been established that Fc-mediated effects are important in immunity dependent on antibodies binding to MSP-1 [15,16], although some studies have shown that this mechanism is not essential [17,18]. One possible explanation for the results is that the IgG3 isotype has a greater capacity to fix complement than IgG2a [19]. Although the FcγRI receptor of macrophages has a high affinity for IGg2a, it binds IgG3 with fivefold higher affinity [20,21]. In competition studies, it has been demonstrated that IgG3 can inhibit IgG2a binding to FcγRI receptor [21]. Earlier work with a different IgG3 specific for MSP-1 also showed that it was effective in passive protection [10]. This interpretation would suggest that IgG3 is the best immunoglobulin sub-class for use in passive immunization to eliminate the parasite from the blood.

Smith and Taylor-Robison [22] measured the level of different immunoglobulin isotypes induced during infections of mice with virulent and avirulent lines of *Plasmodium chabaudi *and *Plasmodium yoelii*. A non-lethal infection was characterized by early upregulation of IgG2a and late upregulation of IgG1, whereas in a lethal infection a slow and reduced IgG2a correlated with rapid fatal outcome before G1 synthesis. Unfortunately these authors did not evaluate the IgG3 isotype.

The proliferation of spleen cells following stimulation with MSP-1_19 _was observed for the group of mice that was protected by passive transfer of B6 and F5 antibodies, and which was similar in magnitude to that induced by the mitogen ConA and was not replicated when the spleen cells came from normal BALB/c mice. Since the passive immunization did not result in sterile immunity it is likely that the infection induced an acquired immune response in these animals. This immune response may have contributed to the observed control of infection, in addition to that resulting from the presence of the B6 and F5 antibodies. In fact, antibodies to MSP-1_19 _were not detected by western blotting in the serum samples from the mice in this group taken after the infection had been cleared, suggesting that all of the passively transferred antibodies had been consumed and other mechanisms might have provided the final clearance of parasites.

## Conclusions

These studies highlight the fact that monoclonal antibodies binding to the C-terminus of MSP-1 can provide protection by passive immunization. However, the outcome of using combinations of protective antibodies, which have both the same or different isotypes and different epitopes, can produce additive or suppressive effects in passive immunization experiments. These observations highlight the complexity of the interaction of antibodies with the surface of the merozoite and may be important in understanding immunity in this experimental mouse model of malaria. Whilst passive immunization with single antibodies may have potential clinical therapeutic application [23], combinations of antibodies would need to be evaluated very carefully. The results are important in the context of malaria vaccine design since stimulation of the appropriate antibody response either by antigen engineering or by use of appropriate adjuvant may be a critical component in vaccine development.

## Competing interests

The authors declare that they have no competing interests.

## Authors' contributions

IE contributed to the performance of the passive immunization experiments and to drafting the manuscript. GP performed the passive transfer experiments and western blotting experiment. BP contributed to analysis of the data and drafting the manuscript. AH contributed to the final preparation of the manuscript. LS contributed to study design, data analysis, performance of the cell proliferation assays and manuscript writing. All authors read and approved the manuscript.
